# Facial emotion recognition of sadness relates to superior parietal gray matter volume in mild cognitive impairment

**DOI:** 10.1007/s00429-026-03215-4

**Published:** 2026-10-17

**Authors:** Rachana Mahadevan, Naomi Kristin Giesers, Thomas Liman, Karsten Witt, Andrea Hildebrandt, Mandy Roheger, Stephanie Rosemann

**Affiliations:** 1 Ambulatory Assessment in Psychology Lab, Department of Psychology, Carl von Ossietzky Universität Oldenburg, Oldenburg, Germany; 2 Department of Human Medicine, University Clinic for Neurology, Carl von Ossietzky Universität Oldenburg, Oldenburg, Germany; 3 Center for Stroke Research Berlin, Charité – Universitätsmedizin Berlin, Charite Platz 1, 10117 Berlin, Germany; 4 German Center for Neurodegenerative Disease (DZNE), Berlin, Germany; 5 Research Center Neurosensory Science, University of Oldenburg, Oldenburg, Germany; 6 Psychological Methods and Statistics, Department of Psychology, Carl von Ossietzky Universität Oldenburg, Oldenburg, Germany; 7 Biological Psychology Lab, Department of Psychology, Carl von Ossietzky Universität Oldenburg, Oldenburg, Germany

**Keywords:** Mild cognitive impairment, Facial emotion recognition, Cortical thickness, Gray matter volume, Structural MRI, Socioemotional functioning, Structural reserve

## Abstract

Mild cognitive impairment (MCI) is associated with early, regionally selective neurodegenerative changes that may affect socioemotional functioning before atrophy becomes widespread. This study examined whether structural brain alterations in MCI relate to facial emotion recognition performance. Sixty adults (30 MCI, 30 healthy controls), aged 50–86 years, completed neuropsychological testing, the Facial emotion composite Task, and structural MRI. Whole-brain voxel-based morphometry revealed no significant gray matter volume differences between groups. However, targeted region-of-interest ROI analyses demonstrated lower right amygdala and bilateral hippocampal volumes in patients with MCI. Cortical thickness analyses identified two regions with lower thickness in MCI: the right superior parietal lobule and the left posterior insula. A significant Group × Sadness interaction indicated that the association between left superior parietal gray matter volume and sadness recognition differed between groups. Follow-up analyses of the extracted values showed a positive association in MCI, although this within-group association did not reach statistical significance, and a significant inverse association in HCs. These findings suggest that early MCI involves selective thinning in association cortices supporting attention and salience processing. The sadness-specific interaction indicates that patients with MCI may rely on preserved superior parietal structure during sadness recognition, a pattern that is consistent with structural reserve accounts, although this interpretation remains speculative given the cross-sectional design.

**Supplementary Information:**

The online version contains supplementary material available at https://doi.org/10.1007/s00429-026-03215-4.

## Introduction

The brain undergoes gradual structural changes with age, influencing a range of cognitive abilities. The rate of decline, however, varies across individuals, with some experiencing more rapid deterioration than others (Perez et al. 2025). When such a decline becomes accelerated, it is often characterized as neurodegeneration, as seen in subtypes of dementia, as well as earlier conditions such as mild cognitive impairment (MCI) and subjective cognitive decline (Dugger and Dickson 2017).

Research has shown that dementia is accompanied by structural brain changes affecting regions such as the hippocampus, entorhinal cortex, frontal cortex, and temporal lobes, depending on the underlying pathology (Arvanitakis et al. 2019; Suri et al. 2014). These structural alterations contribute to progressive cognitive and behavioral decline.

MCI represents a less severe condition than dementia, as individuals at this stage are generally able to carry out their daily activities independently (Gauthier et al. 2006). Longitudinal research indicates that approximately 34% of patients with MCI progress to dementia over time, 25% specifically develop Alzheimer’s disease, around 45% remain clinically stable, and nearly 15% revert to unimpaired cognitive functioning (Hu et al. 2017).

Similar to dementia, structural changes have been reported in several brain regions in MCI, including the parahippocampal cortex, middle and inferior temporal gyri, supramarginal gyrus, superior frontal gyrus, entorhinal cortex, amygdala, fusiform gyrus, and precuneus (Csukly et al. 2016; Del Valdés Hernández et al. 2018; Deschamps et al. 2014; Haussmann et al. 2017; Igarashi 2023; Kobro-Flatmoen et al. 2016; Lau et al. 2023; Ma et al. 2020; Pennanen et al. 2004; Scheff et al. 2011; Tabatabaei-Jafari et al. 2015; Zhu et al. 2020). These alterations have been associated with impairments across multiple cognitive domains, including memory, visuospatial processing, language, face perception, and emotional processing. Furthermore, structural network atrophy has been identified within the temporal, parietal, and orbitofrontal cortices, with pronounced involvement of the medial temporal lobe, fusiform gyrus, and parahippocampal regions (Lau et al. 2023).

While these regions are known to play an important role in cognition, with their structural decline being closely associated with impairments in general cognitive domains such as memory, attention, executive functioning, language, and visuospatial functioning (Albert et al. 2011; Roloff et al. 2025; Wei et al. 2018), research has increasingly extended beyond these domains to examine other affected functions, including emotion recognition and socio-emotional processing (Roheger et al. 2022). Recognizing the emotions of others is a fundamental aspect of social interaction (Ekman and Friesen 1971; Ekman and Oster 1979), and this ability activates a network of brain regions, including the amygdala, insula, fusiform gyrus, primary visual cortex, precuneus, hippocampus (Takashima et al. 2016), posterior superior temporal sulcus, dorsomedial and ventrolateral prefrontal cortices, and supramarginal gyrus (Kim et al. 2015; Schirmer and Adolphs 2017; Spunt and Adolphs 2019). These regions, however, have primarily been identified through functional neuroimaging. Their contribution to emotion recognition, however, depends on the structural integrity of the underlying tissue. Accordingly, structural alterations in these regions, whether reflected in gray matter volume or cortical thickness, would be expected to influence emotion recognition performance. Consistent with this, structural imaging studies have begun to link regional brain morphology with emotion recognition abilities. Gandia-Ferrero and colleagues (2024) reported that lower gray matter volume in regions including the insula, fusiform gyrus, and anterior cingulate cortex was associated with poorer emotion recognition in patients with MCI (Gandia-Ferrero et al. 2024). Similarly, Szymkowicz and colleagues (2016) demonstrated that hippocampal volume was associated with faster facial emotion identification in older adults (Szymkowicz et al. 2016). Additional structural evidence is provided by Padulo and colleagues (2023), who reported amygdala subfield atrophy in individuals showing early neurodegenerative changes associated with MCI (Padulo et al. 2023), and Orlando and colleagues (2023), who related structural brain measures to emotion recognition in HCs (Orlando et al. 2023). Collectively, these studies provide a structural basis for the present investigation.

Since many of these areas show atrophy in MCI, patients are likely to experience deficits in facial emotion recognition. Emotion recognition deficits are well documented in dementia, especially Alzheimer’s disease, but research on MCI remains comparatively sparse (González-Alcaide et al. 2021; Rosen et al. 2004; Shimokawa et al. 2001). The majority of existing studies, however, have reported deficits in emotion recognition in MCI, particularly for negative emotions such as anger, fear, and sadness (Elferink et al. 2015; McCade et al. 2011; Spoletini et al. 2008; Weiss et al. 2008), while happiness recognition remains intact (Barbieri et al. 2022; Elferink et al. 2015). This emotion-specific pattern has been attributed to differences in the neural and cognitive demands of emotion recognition (Fusar-Poli et al. 2009; Lindquist et al. 2012). Recognizing negative emotions relies more heavily on fronto-limbic and temporo-parietal networks, including the amygdala, insula, and cingulate cortex (Dal Monte et al. 2013). These networks are particularly vulnerable to early structural and functional changes in MCI (Talwar et al. 2021; Yang et al. 2012). Furthermore, negative emotions are more ambiguous perceptually and cognitively demanding, requiring the integration of more configural facial cues and greater attentional resources (Bora and Yener 2017; Ruffman et al. 2008). In contrast, happiness is characterized by highly distinctive and salient facial features, which allow for relatively preserved recognition despite broader cognitive and neural decline (Calvo et al. 2008; Ruffman et al. 2008).

Nevertheless, only a limited number of studies (Padulo et al. 2023) have directly investigated the association between structural atrophy and impairments in emotion recognition in MCI. Although emotional processing has often been associated with right-hemisphere specialization (Borod et al. 1998), more recent evidence suggests that hemispheric lateralization is graded and context dependent rather than absolute (Gainotti 2024; Palomero-Gallagher and Amunts 2022). As the present study examined whole-brain structural alterations in MCI rather than hemispheric specialization, we therefore did not formulate hypotheses regarding hemispheric lateralization and instead allowed effects to emerge in either hemisphere.

Based on previous evidence of region-specific vulnerability in MCI and the functional relevance of these regions for emotion processing, we formulated the following hypotheses. First, we hypothesized that individuals with MCI would show lower hippocampal and amygdala volumes compared with HCs (Csukly et al. 2016; Lau et al. 2023; Suri et al. 2014; Tabatabaei-Jafari et al. 2015). Second, we hypothesized that individuals with MCI would show lower cortical thickness of the entorhinal cortex, fusiform gyrus, and cingulate gyrus compared with HCs (Csukly et al. 2016; Lau et al. 2023; Tabatabaei-Jafari et al. 2015; Takashima et al. 2016). Third, we hypothesized that individuals with MCI would exhibit a more widespread pattern of gray matter loss involving the posterolateral and basal temporal lobes, posterior cingulate cortex, anterior insula, and medial frontal lobe compared with HCs (Csukly et al. 2016; Lau et al. 2023; Takashima et al. 2016). Finally, we hypothesized that better facial emotion recognition performance would be positively associated with greater gray matter volume and cortical thickness in brain regions supporting emotion processing. Based on previous evidence, we expected individuals with MCI to show both lower brain volume and poorer facial emotion recognition performance than healthy controls (Elferink et al. 2015; Kim et al. 2015; McCade et al. 2011; Padulo et al. 2023; Schirmer and Adolphs 2017; Spoletini et al. 2008; Spunt and Adolphs 2019; Takashima et al. 2016; Weiss et al. 2008).

## Methods

The study included two main groups of interest, namely, healthy controls (HC) and patients with MCI. We pre-registered our study prior to data acquisition (October 2023) at the Open Science Framework (https://osf.io/yn3gp), and the reporting of this paper follows the TREND statement checklist (Des Jarlais et al. 2004), which can be found in the supplementary section Table S1. The study was approved by the Medical Ethics Committee of the Carl von Ossietzky Universität Oldenburg (Medizinische Ethikkommission; AKZ 2023-062).

### Participant characteristics

**Fig. 1:**
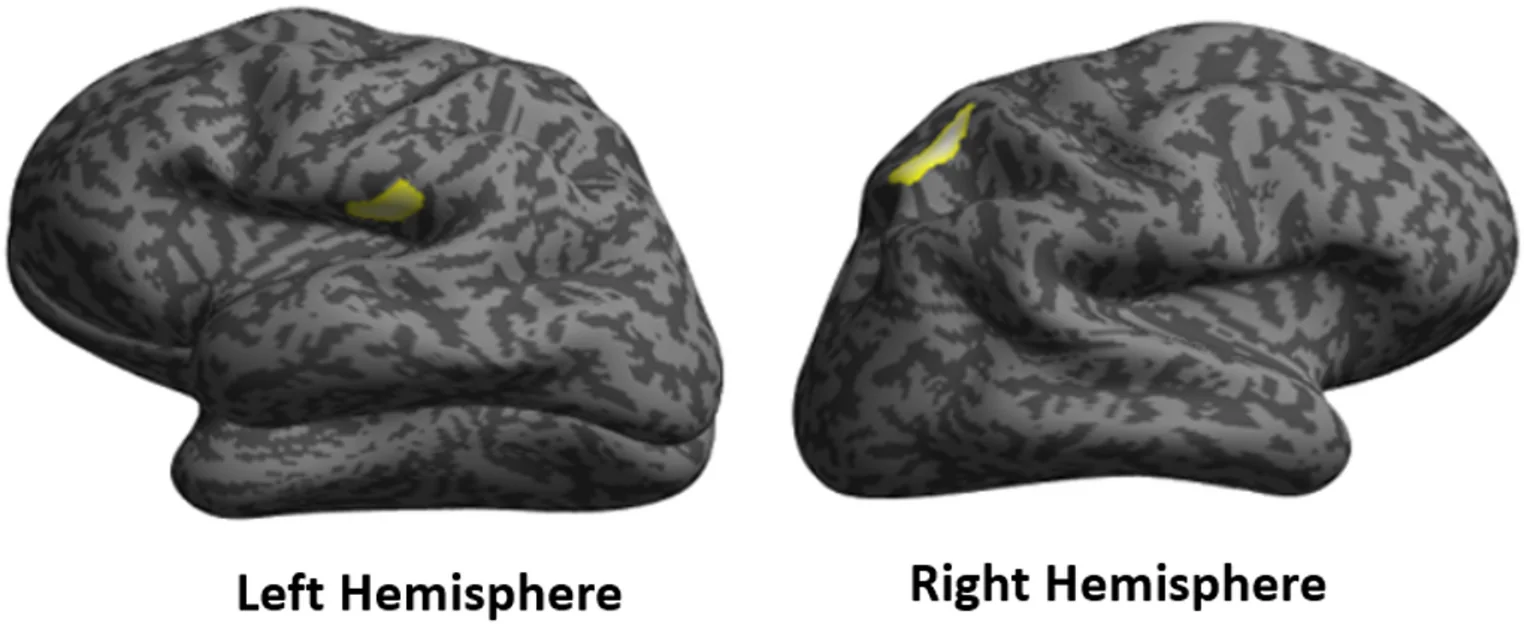
Significantly higher cortical thickness in healthy controls compared to patients with MCI in the left posterior insula (MNI: x=–45, y=–4, z=–6) and the right superior parietal lobule (MNI: x = 23, y=–42, z = 17) [*p* < 0.05; FWE corrected on the cluster level]

This study was planned and conducted at the Department of Psychology, Carl von Ossietzky Universität Oldenburg, Germany, from October 2023 to December 2024. An á priori power analysis conducted before data collection indicated a target sample size of 74 participants. In the early stages of the study, we intended to recruit both patients with amnestic MCI and patients with non-amnestic MCI. However, we were unable to find an adequate number of patients with naMCI within the planned timeframe. Therefore, the current study sample consists of a mix of both subtypes, with a higher number of patients with amnestic MCI (*n* = 25) and only a small number of patients with non-amnestic MCI (*n* = 5). Given the marked imbalance between the amnestic and non-amnestic MCI subtypes, all individuals with MCI were analysed together as a single group. Due to these recruitment constraints, the final sample comprised 60 participants belonging to the age range of 50 to 86 years (*M* = 66.8, *SD* = 8.7), and among them, 30 participants were HCs (age: *M* = 63.1, *SD* = 7.6; 17 women, 13 men, 0 diverse) and 30 patients with MCI (age: *M* = 70.5 *SD* = 8.2; 14 women, 16 men, 0 diverse). Inclusion criteria were: age 50 years or older, fluency in German, functional independence as indicated by a score of 6 or more on the Instrumental Activities of Daily Living questionnaire, and the ability to provide written informed consent. Exclusion criteria were: any diagnosed psychiatric or neurological disorder (e.g., stroke, Parkinson’s disease, epilepsy, multiple sclerosis, traumatic brain injury), substance dependence within the past 6 months, elevated anxiety or depression scores on the Hospital Anxiety and Depression Scale (Zigmond and Snaith 1983), with cut-offs of < 8 for HCs and < 11 for MCI (Ismail et al. 2017), and MRI contraindications such as implanted metallic or electronic devices. Participants in the MCI group were recruited through the Neurology Department of the Evangelisches Krankenhaus Oldenburg following an initial clinical assessment by the treating neurologists. Initial pre-selection was based on clinical suspicion of memory impairment. Final group classification for the present study was established in consultation with the neurology team using the Montreal Cognitive Assessment (Nasreddine et al. 2005). Participants scoring between 18 and 25 were classified as MCI, whereas participants scoring ≥ 26 were classified as HCs. Functional independence was additionally confirmed using the Instrumental Activities of Daily Living questionnaire (Lawton and Brody 1969). Patients with aMCI and naMCI were determined using the CERAD Neuropsychological Assessment Battery (Morris et al. 1989). Patients with *z*-scores below − 1.5 *SD* in one or more memory domains were classified as amnestic MCI, whereas those with impairment confined to non-memory domains (one or more) were classified as non-amnestic MCI. Although CERAD-NAB performance was used to determine MCI subtype, overall group classification (MCI vs. HC) was based on MoCA performance in accordance with the preregistered study criteria. The complete recruitment and exclusion flow for this study is shown in Fig. 1 of the associated manuscript (Mahadevan et al. 2026) and Participant demographic and clinical characteristics are summarized in Table 1.

**Table 1:** Participant demographic and clinical characteristics

Variable	M	SD	MCI (*n* = 30)		HC (*n* = 30)		HC vs. MCI
			M	SD	M	SD	*p*-value
Age	66.80	8.66	70.50	8.09	63.10	7.55	< 0.001
Education	15.60	2.57	15.10	2.85	16.20	2.11	0.090
MoCA	25.40	2.71	23.10	1.55	27.70	1.25	**< 0.001**
ECT	61.40	14.30	58.10	14.20	64.80	13.70	0.071
EI	5.51	0.70	5.27	0.74	5.74	0.58	**< 0.05**
HADS-A	3.18	2.13	3.77	2.19	2.60	1.92	**< 0.05**
HADS-D	1.95	1.95	2.27	2.45	1.63	1.25	0.212

* M*= mean, *SD*= standard deviation, MoCA= Montreal cognitive assessment, ECT=Emotion composite task, EI= Emotional intelligence, HADS-A = Hospital Anxiety and Depression Scale - Anxiety subscale, HADS-D = Hospital Anxiety and Depression Scale - Depression subscale. The mean and standard deviation are displayed along with the p values represent the group comparison between MCI and HCs. Statistically significant results are shown in bold (*p* < 0.001 and *p* < 0.05)

### Procedure

#### Behavioral data acquisition

Our recruitment phase included telephone interviews with checklists that ensured that participants were suitable for our study. All participants signed a written consent form before taking part in the study. The first session was conducted in the lab, where the participants were screened on the IADL (Lawton and Brody 1969) and the HADS (Zigmond and Snaith 1983). The MoCA (Nasreddine et al. 2005) was administered to classify participants as HCs (cut-off score ≥ 26) and patients with MCI (cut-off scores between 18 and 25). Apart from this, the session consisted of tests that measured the participant’s performance in neurocognitive domains such as language, attention, memory, visuospatial construction, and executive functioning via the Consortium to Establish a Registry for Alzheimer’s Disease Neuropsychology battery ( CERAD-NAB; Morris et al. 1989), emotional intelligence via the Trait emotional intelligence questionnaire-short form (Petrides 2009), facial processing via the Facial composite task, and emotion recognition via the facial emotion composite task. The results of these assessments, as well as the associations between the neuropsychological, socioemotional, and face-processing measures and performance on the Facial Emotion Composite Task are reported elsewhere (Mahadevan et al. 2026). The present paper focuses specifically on the relationship between performance on the Facial emotion composite task and structural T1-weighted MRI measures.

#### MRI data acquisition

MRI data was acquired by a 3T whole-body Siemens Magnetom Prisma MRI scanner with a 20-channel head coil at the Neuroimaging unit of the Carl von Ossietzky Universität Oldenburg. Structural images were acquired with a 3-D T1-weighted sequence (MP-RAGE, TR = 2000 ms, TE = 2.07 ms, voxel size = 0.8 × 0.8 × 0.8 mm, flip angle 9 degrees, 224 sagittal slices). During data acquisition, the software of the MRI system was updated from VE11C to XA30. The sequence parameters remained unchanged after the update, as confirmed by a repeated pilot scan. Subsequent to this, data were collected from 18 participants (MCI = 11, HC = 7).

### Materials

#### Facial emotion composite task

Facial emotion recognition was assessed using the Facial emotion composite task from Wilhelm and colleagues (Wilhelm et al. 2014). The task follows the composite-face paradigm, in which the upper and lower halves of the same face display different emotions, and builds on the finding by Calder and colleagues(Calder et al. 2000) that negative emotions (anger, fear, sadness) are primarily conveyed through features of the upper face, whereas positive emotions (happiness, surprise, disgust) are more accurately identified from the lower face. In this task, upper-face expressions were combined with lower-face expressions to create nine composite configurations. The task consisted of six practice trials followed by 144 experimental trials and was implemented in PsychoPy. Each trial began with a fixation cross, after which a cue (UPPER or LOWER) instructed participants to identify the emotion expressed in the corresponding half of the composite face. Responses were made via mouse click from six labelled options, and a brief blank screen separated successive trials. For subsequent correlational analyses with MRI measures, we used percentage accuracy from the Facial Emotion Composite Task (overall and emotion-specific performance).

### Data analysis

To test our hypotheses, we performed the structural MRI analysis in SPM12 and the CAT12 toolbox (Gaser et al. 2024) based on Matlab (2023b; The MathWorks, Inc., Natick, Massachusetts, United States). Voxel-based morphometry (VBM) and surface-based morphometry (SBM) analyses were performed on anatomical T1-weighted images. The data was pre-processed in CAT12 following the standard pipeline including spatial normalization to the Montreal Neurological Institute (MNI) stereotactic space, segmentation into gray matter, white matter, cerebrospinal fluid, and smoothing (Gaussian smoothing, full width at half maximum = 8 mm for VBM gray matter analysis and full width at half maximum = 12 mm for SBM cortical thickness analysis). All datasets underwent quality control for artifacts, motion, and normalization accuracy prior to smoothing. As all participants met these quality criteria, all data were included in the statistical analysis.

Our analysis aimed to examine the group differences on structural measures, and hence we conducted group comparisons using voxel-wise general linear models (GLMs) implemented in SPM12. Analyses were specified using the two-sample *t*-test design within the SPM12 general linear model framework, with age included as a covariate of no interest in all models and total intracranial volume (TIV) additionally included in the VBM analyses to account for individual differences in brain size. The resulting group contrasts (HC > MCI and MCI > HC) were therefore adjusted for these covariates. Additionally, the overall scores of the emotion composite task were included as covariates of interest to assess their association with gray matter volume and cortical thickness. Similarly, subsequent models included the scores of each emotion as covariates. The model specifications were performed in CAT12, and the model estimation and statistical analysis were carried out in SPM12. To examine our a priori hypotheses regarding the hippocampus and amygdala, atlas-based ROI analyses were additionally performed using regional gray matter volumes extracted from the Neuromorphometrics atlas implemented in CAT12. Linear regression models were fitted separately for the bilateral hippocampus and amygdala, controlling for age and TIV. Because four ROIs were tested, *p*-values were corrected for multiple comparisons using the false discovery rate (FDR). For the whole-brain VBM and cortical thickness analyses, statistical significance was assessed at *p* < 0.05 using family-wise error (FWE) cluster-level inference with a cluster-forming threshold of *p* < 0.001. Peak coordinates are reported in MNI space. As a sensitivity analysis, all structural MRI analyses were repeated after excluding the five participants with non-amnestic MCI to assess whether MCI subtype composition influenced the findings.

## Results

### Demographic and behavioral characteristics

There was no significant difference in overall performance on the facial emotion composite task between the groups (HC: *M* = 44.98, *SD* = 9.53; *MCI*: *M* = 40.37, *SD* = 9.88; *t*(57.92) = 1.40, *p* = 0.071, *d* = 0.47). However, for the individual emotions, patients with MCI showed significantly lower performance than HCs for anger (HC: *M* = 65.42, *SD* = 16.16; MCI: *M* = 49.44, *SD* = 18.59; *t*(56.90) = 3.55, *p* < 0.001, *d* = 0.92). No significant differences were observed for sadness (HC: *M* = 25.70, *SD* = 13.60; MCI: *M* = 20.80, *SD* = 15.70; *t*(56.78) = 1.29, *p* = 0.206, *d* = 0.33), fear (HC: *M* = 23.33, *SD* = 13.20; MCI: *M* = 23.47, *SD* = 15.14; *t*(56.95) = − 0.04, *p* = 0.970, *d* = 0.01), happiness (HC: *M* = 60.00, *SD* = 18.00; MCI: *M* = 62.64, *SD* = 14.08; *t*(54.82) = − 0.63, *p* = 0.530, *d* = 0.16), surprise (HC: *M* = 47.92, *SD* = 15.19; MCI: *M* = 45.14, *SD* = 14.56; *t*(57.90) = 0.72, *p* = 0.472, *d* = 0.19), or disgust, although disgust showed a trend toward lower performance in MCI (HC: *M* = 47.50, *SD* = 14.95; MCI: *M* = 40.69, *SD* = 15.35; *t*(57.96) = 1.74, *p* = 0.087, *d* = 0.45).

### Structural brain differences between HCs and patients with MCI

We conducted a whole-brain voxel-based morphometry (VBM) analysis to compare gray matter volume between HCs and patients with MCI while controlling for age and TIV. Group comparisons were performed using the GLM framework described in Sect. Data Analysis. No significant differences between HCs and patients with MCI were detected at the whole-brain level for gray matter volume. To further examine whether subtle alterations in the hippocampus and amygdala may have been obscured by the conservative whole-brain correction, we performed targeted ROI analyses using atlas-based regional gray matter volumes derived from the Neuromorphometrics atlas implemented in CAT12. Linear regression models controlling for age and total intracranial volume revealed significantly lower right amygdala gray matter volume in the MCI group (B = − 0.052, *SE* = 0.018, *t* = − 2.84, *p* = 0.006). Lower right (B = − 0.159, *SE* = 0.062, *t* = − 2.56, *p* = 0.013) and left hippocampal gray matter volumes (B = − 0.157, *SE* = 0.064, *t* = − 2.46, *p* = 0.017) were also observed in the MCI group, whereas no significant difference was detected for the left amygdala (*p* = 0.134). Following FDR correction, the right amygdala and both hippocampal findings remained statistically significant (all FDR-adjusted *p* = 0.023). These findings are summarized in Table 2.

**Table 2:** Atlas-based ROI comparisons between MCI and HC groups

ROI	Hemisphere	b	SE	t(56)	*p*	FDR *p*
Amygdala	Right	−0.0523	0.0185	−2.835	0.006	0.023
Amygdala	Left	−0.0302	0.0199	-1.520	0.134	0.134
Hippocampus	Right	−0.1586	0.0619	−2.564	0.013	0.023
Hippocampus	Left	−0.1574	0.0641	−2.455	0.017	0.023

Negative coefficients indicate lower regional gray matter volume in the MCI group relative to HCs. All models controlled for age and total intracranial volume. *p*-values for the group coefficients were corrected across the four ROIs using the false discovery rate (FDR)

We also conducted a whole-brain SBM cortical thickness analysis between HCs and patients with MCI while controlling for age. Group comparisons were performed using the same GLM framework, controlling for age. In the HC > MCI contrast, significant differences were obtained in two clusters, indicating that HCs showed significantly greater cortical thickness in these regions as compared to the patients with MCI. The first significant cluster was located in the right superior parietal lobule (MNI: x = 23, y=-42, z = 17; *p* = 0.002; cluster size k = 227; z-value = 3.94; Fig. 1), and the second significant cluster was located in the left posterior insula (MNI: x=-45, y=-4, z=-6; *p* = 0.024; cluster size k = 146; z-values = 3.72; Fig. 1). The findings are summarized in Table 3. In the MCI > HC contrast, no significant differences in cortical thickness were obtained.

### Brain structure–behavior associations in facial emotion recognition

**Fig. 2:**
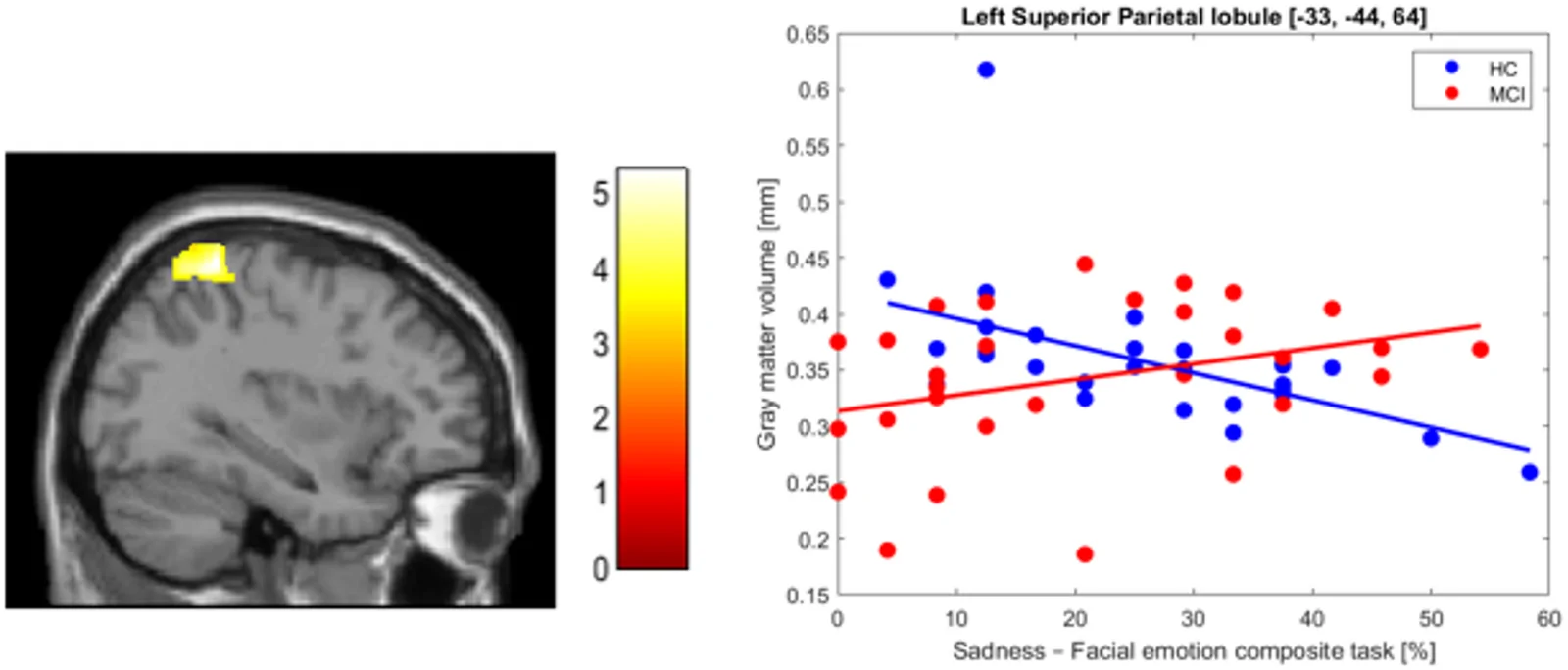
Significant interaction between the gray matter of the left superior parietal lobule (MNI: x = -33, y = -44, z = 64) and the facial emotion composite task performance percentages for sadness. Gray matter volume values extracted from this cluster illustrate opposite directions of association with sadness recognition across groups. The HC association should be interpreted cautiously because regression diagnostics identified influential observations. [*p* < 0.05; FWE corrected on the cluster level]

Next, we tested whether gray matter volume was associated with participants’ facial emotion recognition abilities and whether this association differed between groups. We first added overall facial emotion composite task scores as a covariate in the whole-brain VBM analysis while controlling for age and TIV. No significant group × task interaction effects were found for the overall scores. We then repeated the analysis for individual emotions. For sadness, a significant group × sadness interaction was identified in the left superior parietal lobule (MNI: x = − 33, y = − 44, z = 64; *p* = 0.026; cluster size: k = 918; z = 4.73; see Fig. 2; Table 3). Follow-up regression analyses of the extracted values indicated a positive association in the MCI group, although this within-group association did not reach statistical significance (*b* = 75.20, *SE* = 41.46, *t*(28) = 1.81, *p* = 0.080). In the complete HC sample, the association was negative (*b* = − 121.13, *SE* = 35.53, *t*(28) = − 3.41, *p* = 0.002). Regression diagnostics identified two influential observations in the HC group. After excluding both observations, the negative association remained statistically significant and increased in magnitude (*b* = − 223.09, *SE* = 59.77, *t*(26) = − 3.73, *p* < 0.001). No significant interactions emerged for anger, happiness, disgust, fear, or surprise.

We further examined whether facial emotion recognition abilities were associated with differences in cortical thickness between groups. For this, we conducted a whole-brain cortical thickness analysis, including overall facial emotional composite task scores as a covariate while controlling for age. This analysis did not yield any significant effects. Similarly, when we conducted separate similar analyses for individual emotions (such as anger, sadness, happiness, disgust, fear, and surprise), no significant associations were obtained.

Sensitivity analyses excluding the five participants with non-amnestic MCI yielded the same overall pattern of structural and brain-behavior findings. The significance and direction of the principal findings remained unchanged (see Supplementary Table S2). Sensitivity analyses excluding the single left-handed participant of our sample showed that the pattern and statistical interpretation of the main neuroimaging findings remained unchanged (Supplementary Table S3).

**Table 3:** Significant whole-brain voxel-based and surface-based morphometry findings

Analysis	Effect/Contrast	Region	Hemisphere	MNI coordinates (x, y, z)	Cluster size k	Peak z	FWE-corrected *p*
SBM	HC > MCI	Superior parietal lobule	Right	23, − 42, 17	227	3.94	0.002
SBM	HC > MCI	Posterior insula	Left	−45, − 4, −6	146	3.72	0.024
VBM	Group × Sadness interaction (MCI > HC)	Superior parietal lobule	Left	−33, − 44, 64	918	4.73	0.026

VBM = voxel-based morphometry; SBM = surface-based morphometry; MCI = mild cognitive impairment; HC = healthy control; MNI = Montreal Neurological Institute; FWE = family-wise error. Cluster size represents vertices for SBM and voxels for VBM. The Group × Sadness interaction indicates that the association between gray matter volume and sadness-recognition performance was more positive in the MCI group than in HCs. VBM analyses controlled for age and total intracranial volume, whereas SBM analyses controlled for age. All findings survived cluster-level FWE correction at *p* < 0.05, using a cluster-forming threshold of *p* < 0.001

## Discussion

The present study examined structural brain alterations in MCI and their relationship with facial emotion recognition abilities. We hypothesized that patients with MCI would show (1) lower hippocampal and amygdala volume relative to healthy controls, (2) lower cortical thickness in the entorhinal cortex, fusiform gyrus, and cingulate gyrus, (3) a broader pattern of gray matter loss involving posterior temporal, parietal, insular, and medial frontal cortices, and (4) positive associations between structural integrity and facial emotion recognition performance (as measured by the facial emotion composite task). The results provided only partial support for these hypotheses. Patients with MCI showed significant cortical thinning in the right superior parietal lobule and the left posterior insula compared with controls. Although the whole-brain VBM analyses revealed no significant volumetric differences, targeted ROI analyses identified significantly lower right amygdala and bilateral hippocampal volumes in the MCI group. The only significant brain-behavior finding was a Group × Sadness interaction in the left superior parietal lobule. Follow-up analyses of the extracted values indicated a positive association in MCI, although this within-group association did not reach statistical significance. In HCs, the inverse association remained statistically significant after excluding two influential observations. Nevertheless, its biological significance remains unclear, and the reduced range of gray matter values following the exclusions limits its generalizability. Therefore, the HC association should be interpreted cautiously.

### Preserved whole-brain gray matter volume with regional medial temporal atrophy

Our first hypothesis predicted lower hippocampal and amygdala volume in patients with MCI, while our third hypothesis anticipated a broader pattern of gray matter loss involving the posterior temporal, posterior cingulate, anterior insular, and medial frontal regions. This expectation was based on evidence that MCI frequently involves medial temporal degeneration in regions like the hippocampus and entorhinal cortex, alongside involvement of limbic and salience-network hubs such as the amygdala and insula, and posterior midline and association cortices such as the posterior cingulate and temporo-parietal areas (Csukly et al. 2016; Lau et al. 2023; Suri et al. 2014; Tabatabaei-Jafari et al. 2015). Together, these regions support memory-emotion interactions and integration of relevant information for facial emotion recognition.

The results partially supported these expectations. While the whole-brain VBM analyses did not reveal significant gray matter volume differences, the targeted ROI analyses demonstrated significantly lower right amygdala and bilateral hippocampal volumes in patients with MCI. These ROI findings are consistent with previous studies reporting hippocampal (Jack et al., 1999; Pennanen et al., 2004) and medial temporal atrophy (Whitwell et al., 2007) in MCI and early Alzheimer’s disease. Several differences between studies may account for the absence of significant whole-brain VBM findings. One explanation may be that our sample represents a relatively early stage of MCI, where structural decline is subtle and does not yet reach the threshold of detectability at the whole-brain level (Gafuroglu & Rekik, 2019; Şener et al., 2025; Yue et al., 2025). At this stage, the brain may show only very small or localized changes that occur before any obvious shrinkage is visible, reflecting an early, pre-atrophic phase of neurodegeneration. Another possibility is heterogeneity within the group, as some patients remain stable or revert to normal cognition (Hu et al., 2017), which can obscure group-level differences when only some individuals are progressing toward more severe neurodegeneration. On the other hand, our complementary task-based fMRI study (Mahadevan et al., 2025) showed that despite preserved gray matter volume, patients with MCI already exhibit a selective behavioral impairment in sadness recognition (in this case assessed using holistic facial expressions). Although BOLD responses did not differ significantly between groups, patients with MCI showed qualitatively more restricted activation patterns during emotion processing compared with HCs. This pattern suggests that subtle functional alterations may already be present, even if they do not reach statistical significance at the group level. Cortical thickness may capture subtle structural alterations that are not yet detectable as regional gray matter volume differences, consistent with our finding of cortical thickness alterations in the absence of significant whole-brain VBM effects. Finally, methodological sensitivity may play a role, where VBM may be less sensitive to subregional changes than cortical thickness analyses (Hutton et al., 2009; Hyde et al., 2007; Sheng et al., 2020). The differing findings obtained with VBM and cortical thickness analyses likely reflect the distinct anatomical properties measured by each technique. Gray matter volume, as measured by VBM, is influenced by cortical thickness, cortical surface area, and cortical folding (Winkler et al., 2010), whereas cortical thickness specifically measures the distance between the pial and white matter surfaces. Because these metrics capture partially independent aspects of cortical anatomy, they do not necessarily identify the same structural alterations and may therefore yield different findings even when applied to the same dataset (Hutton et al., 2009; Winkler et al., 2010). In the present study, cortical thickness identified group differences in the right superior parietal lobule and left posterior insula, whereas whole-brain VBM did not. This pattern may indicate that early structural alterations involve selective cortical thinning without proportional reductions in regional gray matter volume, although methodological differences between the two approaches may also contribute. Conversely, the sadness-specific brain–behavior association was observed only in the VBM analysis. This may reflect the fact that regional gray matter volume captures variability related not only to cortical thickness but also to cortical surface area and cortical folding, each of which may contribute to individual differences in emotion recognition. In addition, methodological differences between the two CAT12 pipelines, including the recommended smoothing kernels (Gaser et al., 2024), may also have influenced statistical sensitivity. These findings suggest that VBM and cortical thickness analyses provide complementary rather than contradictory information about structural brain alterations in MCI. Taken together, these findings indicate selective medial temporal differences rather than widespread gray matter volume differences in the present MCI sample.

### Cortical thickness in MCI

Our second hypothesis predicted cortical thinning in the entorhinal cortex, fusiform gyrus, and cingulate gyrus, as these regions are consistently reported to show early cortical thinning in MCI and are critically involved in memory consolidation, facial perception, and emotional appraisals (Csukly et al. 2016; Lau et al. 2023; Tabatabaei-Jafari et al. 2015; Takashima et al. 2016). While thinning in these specific regions was not observed, we did identify significant reductions of cortical thickness in the right superior parietal lobule and the left posterior insula in patients with MCI relative to the HCs.

The superior parietal lobule is a core hub of the dorsal attention network (Lanssens et al. 2020), which supports visuospatial attention and the integration of perceptual features (Corbetta and Shulman 2002; Wojciulik and Kanwisher 1999), especially when visual information requires top-down, goal-directed processing (Behrmann et al. 2004), which are essential processes for decoding facial expressions (Fusar-Poli et al. 2009; Sabatinelli et al. 2011). Subtle impairments in attentional and executive functions are frequently reported in early MCI (Gauthier et al. 2006), and thinning in the superior parietal lobule may therefore reflect early vulnerability within attentional control networks and recent work linking superior parietal cortical thinning to Alzheimer’s disease supports this interpretation (Dickerson et al. 2009).

In addition to the superior parietal cortex, we also observed lower cortical thickness in the left posterior insula in patients with MCI. Reductions of cortical thickness in the insula have been reported consistently across previous MCI studies, suggesting that insular involvement may be a characteristic feature of early neurodegeneration (Yao et al. 2012). The insula is a central hub of the salience network, and cognitive impairment has been associated with both reduced insular thickness and a disruption of the insula’s normal role in coordinating information flow between the default-mode and central-executive networks, with these alterations relating to global cognitive performance (Chand et al. 2017). Within this context, the posterior insula thinning observed in our MCI sample may reflect an additional site of early structural vulnerability, potentially indicating disruption of salience-network integrity before widespread atrophy becomes detectable. The lack of thinning in the expected limbic regions, such as the entorhinal, fusiform, and cingulate cortices, may reflect the relatively early disease stage in our sample or inter-individual differences in how MCI progresses (Gafuroglu and Rekik 2019; Hu et al. 2017; Şener et al. 2025; Yue et al. 2025). At the same time, our results align with previous studies showing that early cortical thinning can often emerge in association cortices rather than limbic regions, reinforcing the idea that cortical thickness captures early structural change, especially in MCI (Dickerson et al. 2009; Machulda et al. 2020; Singh et al. 2006). Taken together with the volumetric results, this pattern suggests that cortical thickness may be a more sensitive marker of early neurodegeneration.

### Sadness-specific brain–behavior associations

Our final hypothesis predicted positive associations between structural changes and overall facial emotion recognition performance, such that lower brain volume would be associated with poorer performance. This expectation was only partially supported. Overall facial emotion composite task scores and most individual emotions did not show significant associations with gray matter volume or cortical thickness. However, we identified one emotion-specific interaction with the group in the left superior parietal lobule. Follow-up analyses of the extracted values showed a positive association pattern between left superior parietal volume and sadness recognition in MCI, although this within-group association did not reach statistical significance, whereas HCs showed a significant inverse association. Notably, despite the absence of group differences in overall emotion recognition performance, and despite comparable sadness recognition accuracy between groups, the structural analyses revealed a sadness-specific interaction in the left superior parietal lobule.

Sadness recognition often depends on relatively subtle facial cues and the integration of distributed facial features (Abbruzzese et al. 2019; Ruffman et al. 2008; Visser 2020). The superior parietal lobe is central to these processes as it supports visuospatial attention, top–down attentional allocation, and feature-based perceptual integration (Corbetta and Shulman 2002; Wojciulik and Kanwisher 1999), particularly when visual signals require goal-directed analysis (Behrmann et al. 2004; Han et al. 2004). Prior work in healthy older adults suggests that structural alterations in the superior parietal lobule may enhance emotion recognition under high perceptual demands, possibly by providing additional attentional support during the processing of difficult emotions (Orlando et al. 2023). In patients with MCI, greater left superior parietal lobule volume showed a positive association with sadness recognition, although this within-group association did not reach statistical significance. This directional pattern may be consistent with structural reserve accounts, in which preserved association cortex may help counteract early declines in specialized emotion-processing regions, although the cross-sectional design precludes firm conclusions (Zeller et al. 2024). In HCs, the inverse association should be interpreted cautiously, as it may reflect normal interindividual variability or incidental covariation rather than a specific brain–behavior mechanism.

In line with previous work, negative emotions are generally more challenging to recognize than positive emotions and place greater cognitive and perceptual demands on the emotion recognition system (Ruffman et al. 2008). An important question is why anger showed a behavioral group difference without a corresponding volumetric association, whereas sadness showed a structural interaction despite comparable behavioral performance. These findings are not necessarily contradictory, because group differences in average performance and group differences in structure-performance associations reflect different effects. In our previous behavioral analysis of this sample, anger recognition was specifically associated with face-processing ability (Mahadevan et al. 2026). Difficulties integrating the facial cues presented in the composite task may therefore partly explain the anger impairment. Anger recognition also relies on a distributed network involving amygdalar, insular, visual, and frontotemporal regions (Haxby et al. 2000; Phillips et al. 2003; Vuilleumier and Pourtois 2007). Subtle alterations across this network could affect performance without producing an association with a single regional gray matter measure that survives whole-brain correction, particularly in a sample of modest size. As discussed above, the sadness interaction may instead reflect a different process involving superior parietal attentional resources. Positive emotions, particularly happiness, are typically well preserved in aging and MCI due to their high perceptual salience (Ruffman et al. 2008), which may reduce the need for compensatory engagement of parietal association cortices and help explain why no structural brain-behavior associations were observed for this emotion (Maki et al. 2013; Moradi et al. 2018).

### Limitations, strengths and future directions

The study also has limitations that should be acknowledged. An a priori power analysis conducted before data collection indicated a target sample size of 74 participants. Due to recruitment constraints, the final sample comprised 60 participants. Post hoc power analyses indicated that the present sample was well powered to detect large between-group effects (*d* = 0.80; power = 0.92), but only moderately powered to detect medium effects (*d* = 0.50; power = 0.61) and had low power for small effects (*d* = 0.20; power = 0.19). Consequently, it may have had limited sensitivity to detect more subtle whole-brain structural differences, particularly in interaction analyses involving behavioral covariates. Recruitment of patients with non-amnestic MCI proved challenging, resulting in a modest imbalance between subtypes that may have reduced sensitivity to subtype-specific effects. However, sensitivity analyses excluding the five participants with non-amnestic MCI yielded the same overall pattern of behavioral, structural, and brain-behavior findings, suggesting that the principal conclusions were robust to MCI subtype composition. Nevertheless, the small number of non-amnestic participants precluded meaningful subtype-specific analyses. In addition, the ROI analyses focused on the hippocampus and amygdala because these structures were specified a priori in our hypotheses and are consistently implicated in MCI. Other brain regions were not examined using an ROI approach and therefore may warrant investigation in future studies. As the study used a cross-sectional design, it cannot address how these structural differences evolve over time. Longitudinal studies will therefore be important to determine whether early alterations in association cortices, particularly the superior parietal lobule, predict disease progression or determine whether the observed association reflects structural reserve, compensatory mechanisms, or other sources of interindividual variability. Accordingly, although the observed sadness-specific interaction survived family-wise error correction, it should be interpreted cautiously and replicated in larger, independent cohorts before firm conclusions regarding its neurobiological significance can be drawn.

Despite these limitations, the study offers several strengths. The combination of a detailed behavioral assessment with whole-brain structural analyses provides a thorough perspective on socioemotional functioning in MCI. In addition, the examination of individual emotions allowed us to identify a specific association between sadness recognition and the left superior parietal lobe, demonstrating the value of emotion-specific analyses when investigating early socioemotional changes. These findings also contribute to our understanding of MCI by suggesting that socioemotional impairment reflects alterations in distributed brain networks extending beyond the medial temporal lobe. The emotion-specific association with sadness further indicates that these impairments are selective rather than generalized, emphasizing the importance of considering individual emotions when studying socioemotional dysfunction in MCI. From a clinical perspective, incorporating emotion-specific assessments alongside traditional cognitive measures may improve the characterization of early social-cognitive changes, although the clinical utility of such approaches requires further investigation.

Future work should clarify which perceptual cues patients with MCI rely on when recognizing subtle sad expressions. Approaches such as targeted eye-tracking or feature-based methods may help to identify whether certain facial cues are consistently overlooked in daily interactions. Another important factor would be to examine whether these perceptual patterns are similar across different subtypes of MCI (amnestic single-domain, amnestic multiple-domain, non-amnestic single-domain, non-amnestic multiple-domain).

## Conclusion

This study examined how brain structure relates to facial emotion recognition in MCI. We found evidence of selective medial temporal alterations, with reduced right amygdala and bilateral hippocampal volumes identified in targeted ROI analyses, despite the absence of significant whole-brain gray matter volume differences. In addition, cortical thinning was observed in the right superior parietal lobule and the left posterior insula, suggesting early structural changes in regions involved in attention and salience processing. Sadness was the only emotion that showed a significant interaction between group and brain structure. Follow-up analyses of this interaction showed a positive association pattern in MCI, although the within-group association did not reach statistical significance, and a significant inverse association in HCs that should be interpreted cautiously. These findings indicate that sadness recognition may rely on different structural resources in MCI than in HCs. The positive directional pattern in MCI may be consistent with the role of superior parietal structure in sadness recognition, although this interpretation remains preliminary. Taken together, these findings suggest that early socioemotional changes in MCI may involve structural alterations in parietal and insular regions, with the superior parietal lobule showing a specific role in supporting sadness recognition.

## Supplementary Information

Below is the link to the electronic supplementary material.

Supplementary Material 1

## Data Availability

Due to ethical restrictions, the data and code supporting the findings of this study are not publicly available. They can be requested from the corresponding author.

## Declarations

### Pre-registration

Our study was preregistered on the Open Science Framework (https://osf.io/yn3gp) prior to data collection on 17 October 2023. A preprint version of this manuscript was published on PsyArXiv Preprints on 24th of January 2026 and is available at https://osf.io/preprints/psyarxiv/6uq4e_v1.
